# Substance Use Disorders Assessed Using the Kreek–McHugh–Schluger–Kellogg (KMSK) Scale in an Urban Low-Income and Predominantly African American Sample of Primary Care Patients

**DOI:** 10.1111/j.1521-0391.2011.00121.x

**Published:** 2011-05

**Authors:** Yi-lang Tang, Lamya Khoury, Bekh Bradley, Charles F Gillespie, Kerry J Ressler, Joseph F Cubells

**Affiliations:** 1 Department of Human Genetics, Emory University School of MedicineAtlanta, Georgia; 2 Department of Psychiatry and Behavioral Sciences, Emory University School of MedicineAtlanta, Georgia; 3 Atlanta VA Medical CenterAtlanta, Georgia; 4 Howard Hughes Medical InstituteChevy Chase, Maryland; 5 Yerkes National Primate Research CenterAtlanta, Georgia

## Abstract

The Kreek–McHugh–Schluger–Kellogg (KMSK) scale was developed to quantify self-exposure to opiates, cocaine, alcohol, and tobacco. The original study was limited by a relatively small sample that was not representative of general clinical populations, and did not include marijuana exposure. For the current study, participants were recruited from primary care outpatient clinics in an urban public hospital. The primary measure was the KMSK scale. The Structured Interview for Diagnosis for DSM-IV (SCID) was used as the “gold standard” for substance dependence diagnoses, and the results of KMSK assessments were evaluated using receiver operator characteristic (ROC) analysis. The sample (n = 439) was predominantly African American (90.6%), with mean age (±SD) of 43.1 ± 12.8 years. ROC analyses found that the optimal cutoff scores for alcohol dependence were the same as suggested previously (11), while they were lower for cocaine dependence (10 vs. 11) and opiate dependence (4 vs. 9). The analysis suggested a cutoff score of 8 for marijuana. The KMSK performed well in the current study as a brief tool for evaluating dependence on alcohol, cocaine, marijuana, and opiates in this nonpsychiatric clinic sample of predominantly poor urban African Americans. *(Am J Addict 2011;20:292–299)*

## INTRODUCTION

The high prevalence of substance use disorders (SUD) themselves,^1^–^3^ the substantial substance-related comorbidities associated with nonsubstance-use psychiatric disorders,^4^–^6^ and the seriousness of the medical and psychiatric consequences of SUD^7^ all point to the need, in both research and clinical practice, for brief and easily implemented screening instruments for detecting and evaluating SUD. A number of SUD screening instruments have been developed, such as the Drug Abuse Screening Test (DAST-10)^8^, ^9^ and the Substance Dependence Screening Questionnaire (SDSQ),^10^ which screen across SUD involving multiple substances, and for all SUD, and the CAGE,^11^ Canterbury alcoholism screening test (CAST),^12^ and Alcohol Use Disorders Identification Test (AUDIT)^13^ for alcohol-use disorders. However, the foregoing instruments focus predominantly on the symptoms or outcomes associated with SUD, rather than exposure to substances. In addition, it is difficult or impossible to generate specific diagnoses from most of the existing screens.

The Kreek–McHugh–Schluger–Kellogg (KMSK) scale was developed by Kreek and colleagues^14^ with the goal of creating a rapid screening instrument that can be used for the assessment of the extent of lifetime alcohol and drug use, and for the identification of dependence. The original instrument quantified self-exposure to opiates, cocaine, alcohol, and/or tobacco, and a subsequent revision added assessment of marijuana use (2004 e-mail from M.J. Kreek to JFC). Each section of the KMSK scale assesses the frequency, amount, and duration of use of a particular substance during the individual's period of greatest consumption. It takes approximately 15–20 minutes to administer the entire instrument. Since its publication, this scale has been used in several genetic studies of drug abuse, mostly by the same group that developed the instrument.^15^–^17^

The original study^14^ showed the KMSK could assess lifetime use of tobacco, alcohol, cocaine, and opiates. However, that study was limited by the relatively small sample size (*n* = 100) and the small number of subjects in each diagnostic group. In addition, the sample evaluated was recruited to participate in studies of SUD, so might not have been representative of more general clinical populations. Finally, the original version of the KMSK did not assess marijuana dependence. The aim of the current study was to validate the utility of the KMSK in a larger sample comprised of inner-city primary care patients. Specifically, we tested the hypothesis that the KMSK would show good sensitivity and specificity for diagnosing substance dependence in clinical research settings by comparing the screening results to diagnoses established by structured interview using the Structured Interview for Diagnosis for DSM-IV Axis-I disorders (SCID-I).^18^ Finally, we examined the performance of a version of the KMSK revised by its originators to include marijuana exposure, which the originally described instrument had not included.

## METHODS

### Subjects and Assessments

Subjects in this study were ascertained as part of the Grady Trauma Project, which is an ongoing molecular genetic study with a primary focus on posttraumatic stress disorder.^19^–^21^ Potential participants were approached by research staff in the primary care and obstetrics-gynecology waiting rooms of the Grady Memorial Hospital General Medical Clinic, in Atlanta, GA. The inclusion criteria were: (1) 18 to 65 years old, male or female; (2) able to give informed consent and willing to participate in interviews and collection of biological materials (saliva and/or blood) for DNA extraction. All enrolled participants gave written informed consent, and the study was approved by the Institutional Review Boards of Emory University and Grady Healthcare System. Subjects were reimbursed for their time and effort in the study.

Subjects who completed a brief screening interview (as described in Gillespie et al., 2009)^21^ were invited to participate in a more extensive evaluation. As described in full detail previously,^21^ subjects who agreed to participate in the more extensive evaluation underwent additional assessments, which included the SCID-I.^18^ At that visit, the participants also completed the KMSK. To address variation in literacy in the study population, the KMSK was read aloud to all participants, and answers recorded by staff. The sections on alcohol, tobacco, cocaine, and heroin/opiates of the KMSK used in this study were identical to that described by Kellogg et al.^14^ An additional section on marijuana, added by the authors of the original instrument (2004 e-mail from M.J. Kreek to JFC), was also included in the current version of the instrument.

### Statistical Analysis

All analyses were performed using SPSS17.0 software. Descriptive statistics on demographics were calculated and expressed in terms of the total number of subjects and percentages of the sample as a function of a particular characteristic.

Based on the original report,^14^ a receiver operating characteristics (ROC) analysis^22^–^24^ was done to determine both the concurrent validity of the KMSK scales as compared to the SCID and to find the best cutoff score for alcohol, cocaine, opiates, and marijuana dependence diagnoses (tobacco was not analyzed because there is no SCID scale for nicotine dependence). From the ROC graph, the levels of sensitivity and specificity for each possible cutoff score and an index of accuracy of discrimination provided by the scale can be determined. In this study, the goal was to find the KMSK cutoff score that best predicted which participants received a dependence diagnosis for the above four types of substances.

As an alternative method for determining diagnostic cutoff scores for dependence diagnoses, we used chi-square analysis to determine the best cutoff score. Presence or absence of dependence was assigned according to each possible KMSK score, for each of the four scales, and these assignments were compared to those determined by SCID interview in a two-by-two contingency table. While the choice of a cutoff score may be influenced by the specific intent of the scale and/or the characteristics of a given population, if those things are not an issue, the cutoff score with the highest chi-square value may well be the best choice.^25^,^26^

Several aspects of the ROC analysis can be used to explain the relationship between a scale and a criterion measure. The one most commonly used is the area under the ROC curve (AUROC curve),^27^ which is an overall measure of the relationship between the scale and the criterion. A score of .5 means a chance relationship, and 1.0 represents a perfect relationship. Scores lower than .5 signify a predictive ability worse than chance.

We recoded the SCID scores based on the DSM-IV criteria as described by Kellogg et al.^14^ Briefly, the SCID requires the endorsement of at least three DSM-IV symptoms in order to receive a diagnosis of alcohol or substance dependence. So, patients who had SCID-I score of three or greater for alcohol, cocaine, opiate, and marijuana dependence were give the criterion score of “1”; and those who received scores of 0, 1, or 2 were recoded as “0” (absent) for that substance. The patients who received an abuse but not a dependence diagnosis for a specific substance also received a “0” score for that dependence diagnosis.

## RESULTS

### Sample Characteristics

A total of 439 subjects had complete data on the demographic form, the KMSK, and the SCID and were included in the current analysis. Table 1 summarizes the demographic characteristics of our sample. The sample was predominantly African American (AA, 90.6%). The mean age was 43.1 years (SD = 12.8). The majority of subjects were female (271/439, 61.7%). There was a significant difference in mean ages of each sex, with males being older on average (*p* < 0.01).

**Table 1:** Demographic characteristics of the samples

Demographic	Total sample (N = 439)	Male (N = 168)	Female (N = 271)
Age (mean ± S.D.)*	43.1 ± 12.8	46.6 ± 10.6	40.9 ± 13.5
Self-identified race/ethnicity	(N = 439)	(N = 168)	(N = 271)
Black	398 (90.7)	150 (89.3)	248 (91.5)
Non-black	41 (9.3)	18 (10.7)	23 (8.5)
Education	(N = 438)	(N = 168)	(N = 270)
Did not complete12th grade	98 (22.4)	31 (18.5)	67 (24.8)
High school graduate	153 (34.9)	59 (35.1)	94 (34.8)
Graduate equivalency diploma	25 (5.7)	9 (5.4)	16 (5.9)
Some college/technical school	99 (22.6)	42 (25.0)	57 (21.1)
Technical school graduate	19 (4.3)	7 (4.2)	12 (4.4)
College graduate or higher	42 (10.0)	20 (11.9)	30 (8.8)
Relationship status**	(N = 437)	(N = 167)	(N = 270)
Single or never married	241 (55.1)	80 (47.9)	161 (59.6)
Married	44 (10.1)	20 (12.0)	24 (8.9)
Divorced	88 (20.1)	50 (29.9)	38 (14.1)
Separated	37 (8.5)	15 (9.0)	22 (8.1)
Widowed	27 (6.2)	2 (1.2)	25 (9.3)
Currently unemployed	347/439 (79.0)	141/168 (83.9)	206/271 (76.0)
Currently receiving disability support	125/437 (28.6)	57/166 (34.3)	68/271 (25.1)
Ever been arrested**	274/438 (62.6)	139/168 (82.7)	135/270 (50.0)
Ever been in jail**	257/438 (58.7)	130/168 (77.4)	127/270 (47.0)
Ever been in prison**	68/435 (15.6)	50/167 (29.9)	18/268 (6.7)
Ever had psychiatric hospitalization	80/435 (18.4)	31/166 (18.7)	49/169 (18.2)

N (%) are shown for each demographic variable;

* *p* < .001 between males and females;

** *p* < .001 between males and females, after controlling for age and race.

### Substance Dependence Related Diagnosis in Our Sample

Based on the SCID interview, approximately half (214/439, 48.7%) of the sample did not meet any lifetime substance dependence diagnosis. Alcohol dependence was the most common substance dependence in this sample (145/439, 33.0%), followed by cocaine dependence (90/401, 22.4%), marijuana dependence (16/396, 4.0%), and opiate dependence (12/432, 2.8%). A total of 3.4% of our samples met the diagnostic criteria for polydrug dependence, defined as meeting DSM-IV criteria for at least two classes of substance.

### Kreek–McHugh–Schluger–Kellogg Total Scores of Each Class of Substance

Table 2 shows the correlation analyses of the KMSK lifetime total scores and SCID-I assessments for different types of substances. There were significant correlations between the total KMSK scores and the SCID scores in alcohol, cocaine, and opiates, but not marijuana.

**Table 2:** Analysis of KMSK lifetime total scores and SCID-I assessments for different types of substances

KMSK subscale	Mean KMSK scale score	Mean SCID score	KMSK-SCID correlation
Alcohol whole sample	8.0 ± 4.6	4.1 ± 2.1	r = .473, *p* < .0001
Alcohol dependence (N = 144)	12.0 ± 1.4	5.1 ± 1.3	r = .289, *p* < .0001
Cocaine whole sample	5.4 ± 6.5	.2±.4	r = .792, *p* < .0001
Cocaine dependence (N = 90)	13.7 ± 2.7	5.4 ± 1.4	r = .235, *p* < .026
Marijuana whole sample	5.5 ± 5.0	2.2 ± 1.8	NS
Marijuana dependence (N = 16)	12.0 ± 2.4	4.0 ± 1.3	NS
Opiate whole sample	5.1 ± 2.0	.9 ± 2.6	r = .741, *p* < .004
Opiate dependence (N = 12)	8.5 ± 3.6	5.5 ± 1.3	r = .584, *p* < .046

### Receiver Operating Characteristics Analysis of Alcohol, Cocaine, Heroin, and Marijuana

**Figure 1:**
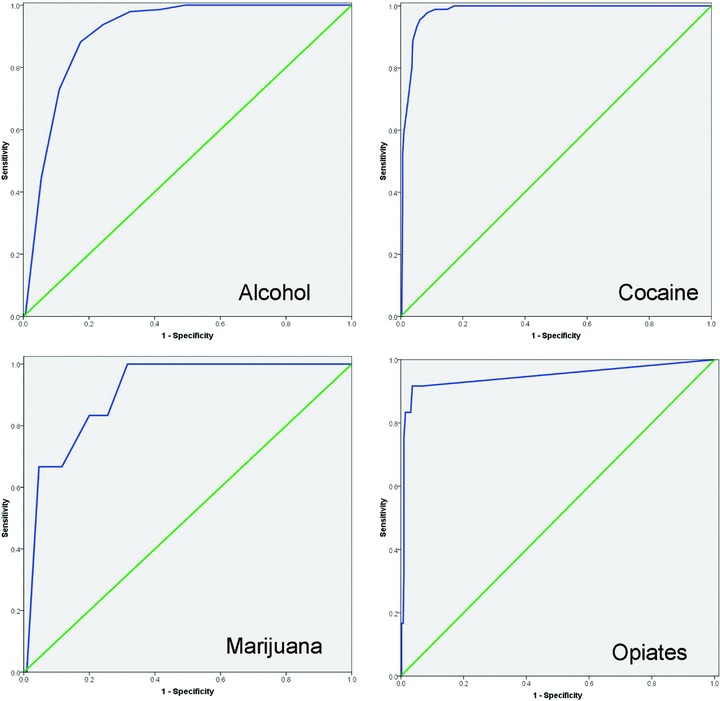
Receiver operating characteristics curves for different substance dependence diagnoses. Axis X is a measure of 1-specificity (false positive rate) of the KMSK scale and Axis Y represents the sensitivity of the KMSK. The diagonal line is a reflection of chance. The specific graphs are: the alcohol dependence ROC graph; the cocaine dependence ROC graph; the marijuana dependence graph; the opiate (heroin) dependence ROC graph.

We performed a series of ROC analyses using the SCID diagnoses as the state variable (coded as 0 and 1, for presence or absence of the diagnosis, respectively), and the total score (lifetime) from each individual substance as the test variable. Figure 1 shows the ROC graphs for alcohol, cocaine, marijuana, and opiate dependence in our sample. We also calculated two other common measures that can show the diagnostic utility of an instrument: the positive predictive potential (PPP) and the negative predictive potential (NPP). The PPP is a measure of the proportion of subjects correctly classified as having the relevant dependence given a specific cutoff score and the NPP reflects how likely the test is to be correct if it categorizes a subject as not having a condition or diagnosis given a specific cutoff score.

### Cutoff Scores for Different Types of Substances

Table 3 shows different cutoff scores, the resultant sensitivity, specificity, chi-square value, PPP, and NPP for alcohol, cocaine, marijuana, and opiates.

**Table 3:** Impact of KMSK cutoff score on sensitivity and specificity

Substance	Cutoff (≥)	Sensitivity (%)	Specificity (%)	Sensitivity + specificity	χ^2^ value	*p* value	PPP	NPP
Alcohol	7	100.0	50.7	150.7	110.5	<.0001	.500	1.000
	8	98.6	58.2	156.8	130.4	<.0001	.538	.988
	9	97.7	67.5	165.2	166.0	<.0001	.597	.985
	10	93.8	75.7	169.5	186.5	<.0001	.655	.961
	11	88.2	82.5	170.7*	199.7*	<.0001	.713	.934
	12	72.9	89.0	161.9	171.8	<.0001	.766	.870
Cocaine	6	98.9	88.9	187.8	251.0	<.0001	.724	.996
	7	97.8	91.5	189.3	271.2	<.0001	.772	.993
	8	96.7	92.5	189.2	276.3	<.0001	.791	.990
	9	95.6	93.8	189.4*	285.7	<.0001	.819	.986
	10	93.3	94.8	188.1	286.8*	<.0001	.840	.980
	11	88.9	96.1	185.0	282.3	<.0001	.870	.967
	12	80.0	96.4	176.4	245.8	<.0001	.867	.943
Marijuana	5	100.0	52.1	152.1	6.34	< .05	.055	1.000
	6	100.0	54.9	154.9	7.07	< .01	.058	1.000
	7	100.0	61.9	161.9	9.32	< .01	.068	1.000
	8	100.0	68.4	168.4*	12.25	<.0001	.081	1.000
	9	83.3	74.4	157.7	9.84	< .01	.083	.994
	10	83.3	80.0	163.3	13.78	<.0001	.104	.994
	11	66.7	88.4	155.1	15.5	<.0001	.138	.990
	12	66.7	92.6	159.3	24.9	<.0001	.200	.990
	13	66.7	95.3	162.0	37.8*	<.0001	.286	.990
	14	33.3	97.2	130.5	15.6	<.0001	.250	.981
Opiates	2	91.7	93.4	185.1	103.3	<.0001	.289	.997
	3	91.7	94.6	186.3	120.8	<.0001	.333	.997
	4	91.7	96.4	188.1*	156.6	<.0001	.423	.997
	5	83.3	96.8	180.1	145.7	<.0001	.435	.995
	6	83.3	97.3	180.6	160.8	<.0001	.476	.995
	7	83.3	98.5	181.8	214.8*	<.0001	.625	.995
	8	75.0	99.0	174.0	214.5	<.0001	.692	.993
	9	75.0	99.0	174.0	214.5	<.0001	.692	.993

* The highest value; PPP: positive predictive potential; NPP: negative predictive potential.

## DISCUSSION

This study extends previously published data on the concurrent validity of the KMSK versus SCID diagnosis for substance dependence.^14^ Based on a larger, more ethnically homogenous sample with higher rates of alcohol and substance dependence, we replicated most of the original findings and uncovered some new findings. We found in general that the KMSK is a useful assessment of alcohol, cocaine, marijuana, and opiate dependence. The scale performed well against the SCID for those substances. This finding supports the conclusion of Kellogg et al.^14^ that the KMSK is a rapid screening instrument that can be used for the assessment of the extent of lifetime alcohol and drug use for the identification of dependence. The ROC analysis determined that the cutoff score that best discriminates between the presence and absence of a DSM-IV diagnosis of alcohol, cocaine, and marijuana dependence were 11, 9, and 8, respectively, in our study sample, while the cutoff scores reported by Kellogg et al.^14^ for alcohol and cocaine dependence were both 11. The score for opiate dependence, however, was inconsistent with the original findings, with our study suggesting a score of 4 or 7, depending on the approach to determining the cutoff, and the Kreek et al. study suggesting a cutoff of 9.

While this study showed that KMSK is a suitable, brief tool to characterize an individual's dependent status on alcohol, cocaine, and opiate, further study is necessary to determine its usefulness in assessing marijuana dependence.

In practice, there are two common ways of determining a cutoff score for a screening scale. One way is to maximize both sensitivity and specificity, which means the cutoff score has to have the best trade-off between sensitivity and specificity^28^; another way is to choose a score that can best discriminate between the presence and absence of a diagnosis made by the “gold standard” method, that is, choosing the score with the highest chi-square value in a 2 × 2 goodness-of-fit test. The latter one was used by the original validation study by Kellogg et al.^14^

### Receiver Operating Characteristics Analysis for Different Dependence Diagnoses

The AUROC curve is commonly used as a summary measure of diagnostic accuracy and it can be interpreted as the probability that a randomly selected diseased case will be regarded with greater suspicion (in terms of its rating or continuous measurement) than a randomly selected nondiseased case. So, based on our data, an AUROC of .981 for cocaine dependence implies that there is 98.1% likelihood that a randomly selected diseased case will receive a more suspicious (higher) rating than a randomly selected nondiseased case. Based on this measure, the KMSK did very well on cocaine, alcohol, marijuana, and opiate dependence.

### Cutoff Score for Marijuana Dependence

Based on the ROC analysis, a cutoff score of 8 would have a sensitivity of 100% and a specificity of 68.4%, yielding the highest sum of sensitivity and specificity. The original report did not report a cutoff score on marijuana, probably due to the small number of subjects who met the criteria of marijuana dependence in their samples (One case qualified for a marijuana dependence diagnosis while another met the criteria for marijuana abuse). Despite the limited number of positive cases, we calculated cutoff scores for marijuana dependence because this is the first study to report a cutoff score for marijuana dependence (Marijuana was not included in the original version of the instrument). Validation of marijuana dependence cutoffs in additional samples is clearly necessary, especially since the correlations between the KMSK and SCID scores for marijuana were the only ones that were not statistically significant. Though the actual reasons for the lack of significance are unclear, the relatively small number of positive cases with marijuana dependence limited our power to detect such a relationship. In addition, it could also mean that the KMSK and SCID capture different aspects of marijuana use. This again shows that further study is needed.

### Cutoff Score for Opiate Dependence

In contrast to other substances, the cutoff scores for opiate dependence determined by different approaches were widely disparate. As shown in Table 3, a cutoff score of 4 was best if we chose it based on maximizing sensitivity (91.7%) and specificity (96.4%). However, if we chose the cutoff score with the highest chi-square value, it would be 7, which had substantially lower sensitivity (83.3%) but only marginally improved the specificity (98.5%). Accordingly, the PPP and NPP changed from .423 and .997 when the cutoff was 4 to .625 and .995, respectively, when the cutoff was 7. The authors would recommend using 4 as a cutoff score for screening purposes (ie, if the KMSK is to be followed with more detailed assessment), when sensitivity should probably be regarded as more important in order to decrease the risk of false negatives. However, in situations where the KMSK is the only instrument used for assessment of SUD, the higher cutoff of 7 is likely to be better, as a substantial improvement in PPP is achieved with only a slight decrement of NPP. Review of the data reported by Kellogg et al.^14^ shows that sensitivity (100%) and specificity (95%) based on cutoff score 4 were both excellent, the change of cutoff score from 4 to 9 only yielded minimal change in specificity (from 95% to 99%), but the authors chose 9 (determined by the chi-square value).

Detailed structured diagnostic instruments such as the SCID, or semi-structured instruments such as the Semi-structured Interview for the Assessment of Genetics of Alcoholism (SSAGA)^29^ or the Semi-structured Assessment for Drug Dependence and Alcoholism (SSADDA)^30^ can provide substantially more detailed information than the KMSK about the patterns of drug use and drug-related problems that underlie dependence. However, for the purpose of identifying people that are likely to qualify for a diagnosis of alcohol and drug dependence, the KMSK is a valid and viable adjunct to the SCID or other more detailed structured interviews, especially in clinical settings. In addition, in some research settings, such as large epidemiological studies or studies where the assessment burden regarding nonsubstance use phenotypes is already high, it can be advantageous to use a briefer instrument. In such settings, the KMSK is a good candidate for assessing lifetime SUD. The issue of trading brevity and simplicity for detail may become a very important one in genetic epidemiological studies, as the magnitude of sample sizes required to detect associations of genome-wide significance is very large.

Of note, we here only report the data on lifetime exposure and this paper does not include current exposure assessment. With the help of the original authors (2004 e-mail from M.J. Kreek to JFC), our team is currently collecting data on current (past 30-day) alcohol and drug exposure and we hope that in the future we could expand the utility of the KMSK to include current dependence diagnoses.

Although the results of the current study are generally encouraging, caution is recommended because of the study's limitations. (1) The results are sample dependent because the measurement properties of the KMSK vary according to patient populations. Results may differ for samples from different populations, and the optimal cutoff scores for the KMSK could change. Therefore, it is important to replicate the findings in other samples, including large community samples. (2) The generalizability of the findings may be limited by the recruitment methodology given that participants were selected for an ongoing molecular genetic study of posttraumatic stress disorder; however, it is important to note that all subjects were approached in a randomized fashion from general medical clinic waiting rooms, regardless of traumatic, psychiatric, medical, or substance use histories. (3) Furthermore, although we did investigate the differences in KMSK cutoff scores between male and female subjects, differences associated with other subgroups, such as age groups or subjects with different education levels, were not conducted given that the sample size was not large enough for such analyses.

In conclusion, this study has demonstrated that the KMSK is a suitable, brief tool that can be used to characterize an individual's dependence on alcohol, cocaine, marijuana, and opiates, at least in the primarily African American, low socioeconomic status, urban population examined in this study. Compared with the original report, we found a diagnostic cutoff score for alcohol identical to that suggested by the original study, but lower cutoffs for cocaine dependence (9 vs. 11 recommended previously) and opiate dependence (4 vs. 9). In the meantime, we also determined a cutoff score for marijuana dependence (8) based on our data, which is a new finding. Since this was the first study to report a cutoff score for marijuana, further validation is clearly necessary. Optimal diagnostic cutoff scores for the KMSK may vary depending on sample demographics, but our results suggest the potential utility of the KMSK for evaluating SUD in diverse populations. Additional studies, seeking to validate the KMSK in other clinical samples, as well as in representative community samples, would greatly enhance the utility of the KMSK as a general research and clinical tool. The instrument might be particularly useful in situations in which brevity and simplicity of administration are necessary, such as large epidemiological studies, or as in the case of the present parent study, where the scientific focus is not substance use, but where SUD comorbidity is expected to be substantial, so that data on substance use are very important.

## References

[1] Kessler RC, Chiu WT, Demler O (2005). Prevalence, severity, and comorbidity of 12-month DSM-IV disorders in the National Comorbidity Survey Replication. Arch Gen Psychiatry.

[2] Degenhardt L, Chiu WT, Sampson N (2008). Toward a global view of alcohol, tobacco, cannabis, and cocaine use: Findings from the WHO World Mental Health Surveys. PLoS Med.

[3] Kessler RC, McGonagle KA, Zhao S (1994). Lifetime and 12-month prevalence of DSM-III-R psychiatric disorders in the United States. Results from the National Comorbidity Survey. Arch Gen Psychiatry.

[4] Kessler RC (2004). The epidemiology of dual diagnosis. Biol Psychiatry.

[5] Glantz MD, Anthony JC, Berglund PA (2009). Mental disorders as risk factors for later substance dependence: Estimates of optimal prevention and treatment benefits. Psychol Med.

[6] Merikangas KR, Mehta RL, Molnar BE (1998). Comorbidity of substance use disorders with mood and anxiety disorders: Results of the International Consortium in Psychiatric Epidemiology. Addict Behav.

[7] Dickey B, Normand SL, Weiss RD (2002). Medical morbidity, mental illness, and substance use disorders. Psychiatr Serv.

[8] Martino S, Grilo CM, Fehon DC (2000). Development of the drug abuse screening test for adolescents (DAST-A). Addict Behav.

[9] Skinner HA (1982). The drug abuse screening test. Addict Behav.

[10] Vazquez FL, Blanco V, Lopez M (2007). Performance of a new substance dependence screening questionnaire (SDSQ) in a non-clinical population. Addict Behav.

[11] Mayfield D, McLeod G, Hall P (1974). The CAGE questionnaire: Validation of a new alcoholism screening instrument. Am J Psychiatry.

[12] Elvy GA, Wells JE (1984). The Canterbury alcoholism screening test (CAST): A detection instrument for use with hospitalised patients. N Z Med J.

[13] Bohn MJ, Babor TF, Kranzler HR (1995). The alcohol use disorders identification test (AUDIT): Validation of a screening instrument for use in medical settings. J Stud Alcohol.

[14] Kellogg SH, McHugh PF, Bell K (2003). The Kreek-McHugh-Schluger-Kellogg scale: A new, rapid method for quantifying substance abuse and its possible applications. Drug Alcohol Depend.

[15] Proudnikov D, Hamon S, Ott J (2008). Association of polymorphisms in the melanocortin receptor type 2 (MC2R, ACTH receptor) gene with heroin addiction. Neurosci Lett.

[16] Levran O, Londono D, O’Hara K (2008). Genetic susceptibility to heroin addiction: A candidate gene association study. Genes Brain Behav.

[17] Mitchell JM, Fields HL, White RL (2007). The Asp40 mu-opioid receptor allele does not predict naltrexone treatment efficacy in heavy drinkers. J Clin Psychopharmacol.

[18] First M, Spitzer R, Gibbon M (2002). *Stuctured Clinical Interview for Axis I DSM-IV-TR Axis I Disorders: Non-patient Edition (SCID-I/NP, 11/2002 revision)*.

[19] Binder EB, Bradley RG, Liu W (2008). Association of FKBP5 polymorphisms and childhood abuse with risk of posttraumatic stress disorder symptoms in adults. JAMA.

[20] Bradley RG, Binder EB, Epstein MP (2008). Influence of child abuse on adult depression: Moderation by the corticotropin-releasing hormone receptor gene. Arch Gen Psychiatry.

[21] Gillespie CF, Bradley B, Mercer K (2009). Trauma exposure and stress-related disorders in inner city primary care patients. Gen Hosp Psychiatry.

[22] Baldessarini RJ, Finklestein S, Arana GW (1983). The predictive power of diagnostic tests and the effect of prevalence of illness. Arch Gen Psychiatry.

[23] Murphy JM, Berwick DM, Weinstein MC (1987). Performance of screening and diagnostic tests. Application of receiver operating characteristic analysis. Arch Gen Psychiatry.

[24] McFall RM, Treat TA (1999). Quantifying the information value of clinical assessments with signal detection theory. Annu Rev Psychol.

[25] Gavin DR, Ross HE, Skinner HA (1989). Diagnostic validity of the drug abuse screening test in the assessment of DSM-III drug disorders. Br J Addict.

[26] Ross HE, Gavin DR, Skinner HA (1990). Diagnostic validity of the MAST and the alcohol dependence scale in the assessment of DSM-III alcohol disorders. J Stud Alcohol.

[27] Bradley KA, Boyd-Wickizer J, Powell SH (1998). Alcohol screening questionnaires in women: A critical review. JAMA.

[28] Olden M, Rosenfeld B, Pessin H (2009). Measuring depression at the end of life: Is the Hamilton Depression Rating Scale a valid instrument?. Assessment.

[29] Bucholz KK, Cadoret R, Cloninger CR (1994). A new, semi-structured psychiatric interview for use in genetic linkage studies: A report on the reliability of the SSAGA. J Stud Alcohol.

[30] Pierucci-Lagha A, Gelernter J, Feinn R (2005). Diagnostic reliability of the semi-structured assessment for drug dependence and alcoholism (SSADDA). Drug Alcohol Depend.

